# Imaging Findings and Clinical Features of Primary Spinal Epithelioid Hemangioendothelioma

**DOI:** 10.3389/fonc.2022.713947

**Published:** 2022-05-30

**Authors:** Pan Liang, Bing-Bing Zhu, Xiu-Chun Ren, Jian-Bo Gao

**Affiliations:** Department of Radiology, The First Affiliated Hospital of Zhengzhou University, Zhengzhou, China

**Keywords:** epithelioid hemangioendothelioma, bone, tomography, x-ray computed, magnetic resonance imaging, diagnosis

## Abstract

**Rationale and Objectives:**

Primary spinal epithelioid hemangioendothelioma (SEHE) is an extremely rare angiocentric vascular neoplasm with inherent metastatic potential, which pursues a clinical course intermediate between hemangioma and angiosarcoma. The present study sought to present computed tomography (CT) and magnetic resonance imaging (MRI) findings and clinical features of primary SEHE and review the literature.

**Materials and Methods:**

Clinical data of four patients with primary SEHE confirmed by pathology from January 2011 to May 2020 were analyzed retrospectively. Two cases underwent CT scan, while 3 cases underwent MRI scan. Image observation included the tumor location, bone changes, morphology, density/signal characteristics, and enhanced features.

**Results:**

The four patients were all women who ranged in age from 25 to 74 years. Four cases of the lesions were in the vertebral body; among them, two cases involved the accessory of the vertebra. Soap bubble appearance was detected in 2 cases, honeycomb osteolytic appearance in 2 cases, a sclerotic margin in 3 cases, and accompanying vertebral compression fracture in 1 case. CT showed a slightly inhomogeneous low density with punctate high-density foci. MRI showed an inhomogeneous low signal on T_1_-weighted image (T_1_WI) and a high signal on T_2_WI. Contrast-enhanced CT/MRI demonstrated moderate to intensive inhomogeneous enhancement of the lesions. The discs were normal. In one case, lesions presented a dural tail sign.

**Conclusions:**

Primary SEHE is so rare in the clinic as an aggressive vascular tumor. The relatively typical clinical features and radiographic findings can help in preoperative diagnosis.

## Introduction

Epithelioid hemangioendothelioma (EHE) is an extremely rare angiocentric vascular neoplasm with inherent metastatic potential, which pursues a clinical course intermediate between hemangioma and angiosarcoma, and this was first described in 1982 by Weiss and Enzinger (1). Most of the EHE cases occur in the soft tissues, while a wide variety of sites may also be affected, including the lung, pleura, spleen, heart, liver, and bone (2). The malignancy behavior of EHE varies between hemangiomas and conventional angiosarcomas (3). There is no standard treatment for this tumor. Surgical excision with negative resection margins followed by external beam irradiation is commonly used as a widely accepted procedure for this tumor (3).

Primary EHE rarely involves the spine; fewer cases of EHE of the spine have been reported in the English literature. To our knowledge, most of the literature is about the pathology and clinical manifestation (3, 4); therefore, it is difficult to systemically define the radiological appearance for these patients. From January 2011 to May 2020, four cases of primary spinal epithelioid hemangioendothelioma (SEHE) were identified from a series of 10,080 patients with bone tumors who underwent treatment. The purpose of the present study was to report our experience with four patients suffering from primary EHE of the spine. The clinical features and radiographic findings are described.

## Materials and Methods

### Subjects

The institutional review board approved this retrospective study, and the requirement for written informed consent was waived.

### Patients

From January 2011 to May 2020, the available clinical records and radiologic imaging studies were reviewed using the following search terms: “Epithelioid hemangioendothelioma” and “hemangioendothelioma.” A total of four cases of primary SEHE were enrolled in the study. All patients had undergone treatment. Clinical data, including age, sex, and symptoms, were obtained.

### CT and MRI

One patient underwent a plain CT scan only, and one patient underwent a plain CT scan and enhanced CT scan. CT images were performed using a 64-multidetector scanner (Discovery CT750HD, GE Healthcare, WI, USA). CT scan (120 kV, 200 mA, bone algorithm reconstruction, and reconstruction section thicknesses 5 mm) was performed before and after the bolus injection of contrast agent (iopromide, 370 mg/ml; GE Medical Systems). The volume of contrast used was calculated as 1.5 ml/kg body weight, and the injection rate was 5 ml/s.

One patient underwent a plain MR scan only, and two patients underwent a plain MR scan and enhanced MR scan. MR images were performed using a 1.5-T unit scanner (GE Signa) and a 3.0-T unit scanner (Philips) by using a receive-only synergy spine coil. Each study consisted of axial and sagittal T1-weighted (TR/TE, 9/420 ms) and T2-weighted (repetition time (TR)/echo time (TE), 100/3,000 ms) images for the sagittal and T2-weighted (TR/TE, 106/3,000 ms) images for the axial images. Section thickness was 5 mm. The enhanced scan was performed after intravenous injection of Gadopentetic acid (Gd-DTPA) (10–15 ml and 2 ml/s).

### Imaging Analysis

The images were reviewed by two experienced radiologists in bone. All analyses were performed at the AW4.6 workstation (GE Healthcare, Waukesha, WI, USA). The reviewers were blinded to the clinical information of the study. The images were evaluated for the tumor location, bone changes, morphology, density/signal characteristics, and enhanced features. The pattern of the bone destruction was classified as soap bubble appearance or honeycomb osteolytic appearance.

## Results

### Patient Characteristics

The four patients were all women who ranged in age from 25 to 74 years (**Table 1**). The clinical and radiological features were summarized in **Table 1**. All patients had non-specific symptoms, including local pain, swelling, pathologic fracture, or burning sensation.

**Table 1:** The clinical and radiological features of 4 primary SEHE patients.

No.	Gender	Age, years	Location	Metastasis site	The main symptoms	Treatment/Diagnosis	Immunohistochemistry
1	Woman	42	Sacral 2–4 vertebrae	No	Burning sensation on the right hip for 3 months	Radical resection	Vimentin (+), CD34 (+), SMA (Blood vessel+)
2	Woman	27	Lumbar 5-sacral 1 vertebra and accessories	No	Swelling and pain in waist and right lower limb for more than 1 year	Biopsy	CD34 (Blood vessel+), EMA (Focal+), S-100 (Focal+), Vimentin (+), ki-67 (25%+), Desmin (Focal+), FIi-1 (+)
3	Woman	25	Lumbar 3 vertebral body and accessories	No	Waist pain for 10 days	Tumor resection, bone grafting and internal fixation	CD34 (+), CD31 (+), ki-67 (1%+), F-VIII (+)
4	Woman	74	Lumbar 3–4 vertebrae	Liver; trunk bones; limb bones; vertebral body	Low back pain for 1 year, worsening for more than half a month	Percutaneous vertebral body cement forming, pathological biopsy	CD34 (+), CD3 1(+), ki-67 (5%+), ERGI (+)

SEHE, spinal epithelioid hemangioendothelioma; SMA, smooth muscle actin; EMA, epithelial membrane antigen.

On immunohistochemical examination, CD34 (100%) was the most frequently positive in these cases, followed by CD31 (50%). The epithelioid cells were positive for vimentin (50%). The morphology and immunohistochemical staining pattern support the diagnosis of primary SEHE.

### CT and MRI Findings

**Figure 1:**
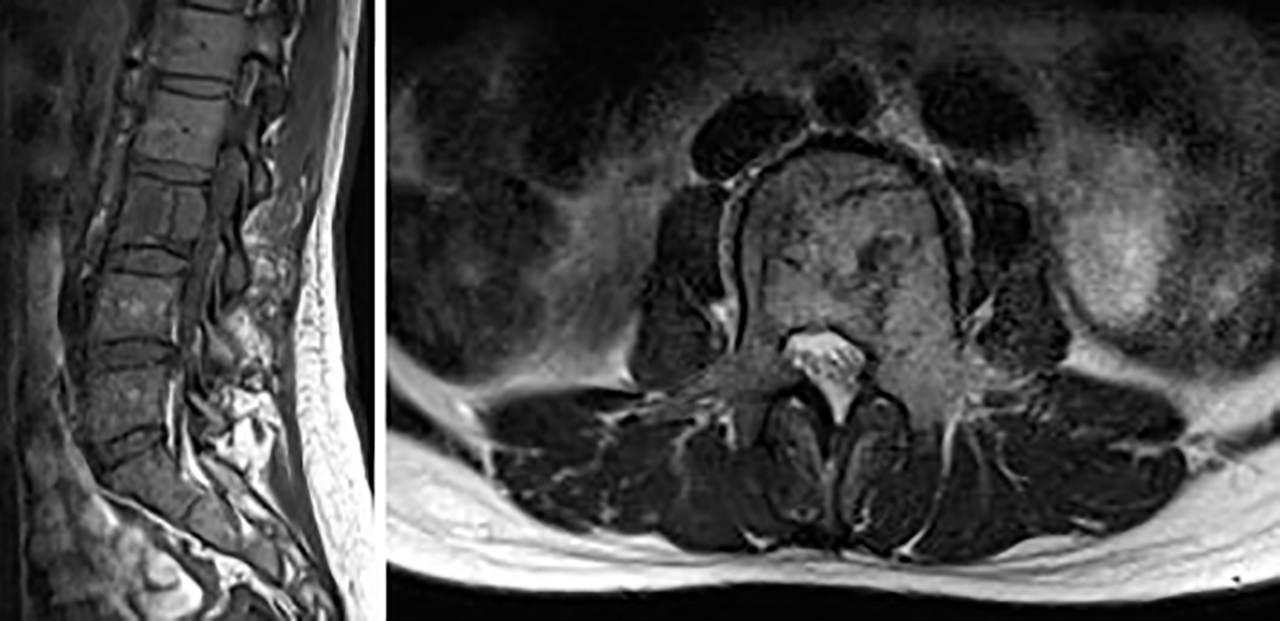
Primary spinal epithelioid hemangioendothelioma (EHE) in a 25-year-old woman. A sagittal image of the lumbar spine reveals the abnormal shape of lumbar 3 vertebral body with a mass in the vertebral body and accessories. MRI showed an inhomogeneous low signal on T_1_-weighted image (T_1_WI) and a high signal on T_2_WI with punctate low-signal foci and accompanying normal discs.

**Figure 2:**
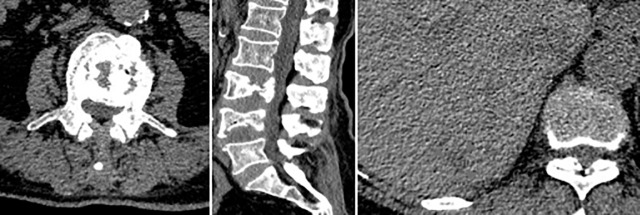
Primary spinal epithelioid hemangioendothelioma (EHE) in a 74-year-old woman. A sagittal image of the lumbar spine reveals a mass that has soap bubble bone destruction in the vertebral body with a secondary vertebral compression fracture. Liver metastasis was demonstrated with an irregular outer layer (arrow).

**Figure 3:**
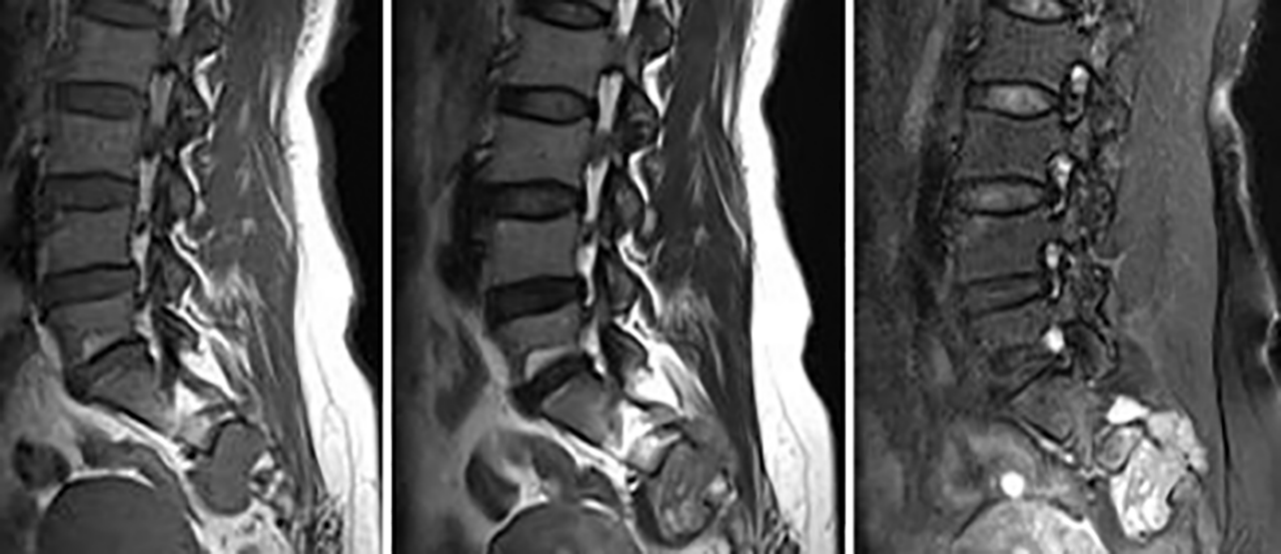
Primary spinal epithelioid hemangioendothelioma (EHE) in a 42-year-old woman. A sagittal image of the lumbar spine reveals a mass in the sacral 2–4 vertebrae with a paravertebral soft tissue mass. MRI showed an inhomogeneous low signal on T_1_-weighted image (T_1_WI) and a high signal on T_2_WI with punctate low-signal foci. Contrast-enhanced MRI demonstrated intensive inhomogeneous enhancement of the lesions with punctate low-signal foci.

One case of the lesion was in the sacral vertebra, one case involved the sacral and lumbar spine, and two cases involved the lumbar spine. Four cases of the lesions were in the vertebral body; among them, two cases involved the accessory of the vertebra. Soap bubble appearance was detected in 2 cases, honeycomb osteolytic appearance in 2 cases (**Figure 1**), a sclerotic margin in 3 cases, and accompanying vertebral compression fracture in 1 case (see arrows on **Figure 2**). CT showed a slightly inhomogeneous low density with punctate high-density foci. One case demonstrated moderate inhomogeneous enhancement of the lesions accompanied by a paravertebral soft tissue mass. MRI showed an inhomogeneous low signal on T_1_-weighted image (T_1_WI) and a high signal on T_2_WI with punctate low-signal foci. Contrast-enhanced MRI demonstrated moderate to intensive inhomogeneous enhancement of the lesions with punctate low-signal foci. Dural tail signs and spinal stenosis were detected in 1 case each. Paravertebral soft tissue mass was detected in 1 case with accompanying normal discs (see arrows on **Figure 3**).

### Follow-Up and Survival

We report two deaths at 21 and 12 months’ follow-up for the disease (case 1 and case 4, respectively; **Table 1**); one out of four patients is presently alive without evidence of disease (case 3). In the second case, due to the change of contact information, we have not been able to contact the patient or her family members.

## Discussion

EHE is an intermediate-grade vascular tumor arising from the vascular endothelium (5). It has been demonstrated that the tumor cells are positive for vimentin, endothelial markers (2), suggesting that EHE of the bone expresses endothelial markers (6). Over 38% of the tumor has been proven to be reactive for cytokeratin and can also focally express the epithelial membrane antigen (6). Due to the rarity of this disease, the possible mechanism of EHE is currently unclear; several cases of cytogenetic studies linked to gene mutations have been reported (7, 8).

Boudousquie et al. (9) reported a patient with EHE with pulmonary and bone marrow metastases, confirming that soft-tissue EHE consists of a complex unbalanced translocation of t (7, 10). Errani et al. (11) demonstrated that the presence of WWTR1-CAMTA1 fusion may serve as a useful molecular diagnostic tool in the diagnosis of EHE. Antonescu et al. (12) reported a novel YAP1-TFE3 fusion in a distinctive subset of EHE of varying sites, the presence of TFE3 immunostaining, and the absence of the more typical t (1, 3) translocation.

Tumors rarely occur in the bone, with an incidence of 1% of all malignant tumors of the bone (2). To our knowledge, there were about fewer original spinal cases reported in recent years. The findings of the present study were consistent with those in the literature. From January 2011 to May 2020, 10,080 bone carcinoma cases were surgically treated at the First Affiliated Hospital of Zhengzhou University. Of these cases, only 4 cases occurred in the spine (0.04%). The age range of the reported cases of primary SEHE was 20–40 years, with no gender predilection, which has an indolent course; some have survived for decades with multiorgan disease (3). In addition, a study about EHE of the bone suggested a slightly male predominance, with a ratio of men to women of approximately 31:25 (6), which is inconsistent with the general trend of the present study. Witte et al. (13) reported that EHE seems to exhibit hormone dependence, responsible for the occurrence of multiple tumor manifestations during pregnancy. In the four cases presented here, the present study did not find similar results. The clinical symptoms of EHE are varied including no obvious early symptoms and local pain, swelling, or pathologic fracture (3). Local pain is common because of the irritation or compression of sinus vertebrae and nerve roots depending on the location of the mass. Three patients (3/4, 75%) in the study presented with local pain consistent with those reported in prior studies (14). In addition, one patient of primary SEHE presented with a burning sensation on the right hip. It is worth mentioning that mostly bone EHE (>50% of cases) is often a multicentric process and the tumor can involve multiple bones or even multiple tissues (2, 6). According to published studies, 20%–30% of EHEs presented distant metastases, and 10%–20% of these metastases were the primary cause of mortality (3, 4, 15, 16).

The CT and MRI appearance diagnosed as EHEs in the spine has not been systematically reported previously. In the present study, the lesions were described as a mass that has an expansile osteolytic lesion with or without a complete or incomplete hardened edge. Ma et al. (5) reported that the EHEs of the spine appeared lytic with no matrix formation and had disruption of the posterior cortex of the vertebral body. It has a distinctive soap bubble matrix with a sclerotic margin like that found in benign vascular tumors, with a residual intact/incomplete bone crest (17). Lesions with ill-defined margins and marked loss of trabeculae are considered more aggressive (18). The tumor with the expansion of bone and possibly an extension through the cortex to adjacent soft tissues is also frequent (5, 17). Osteolytic bone destruction may be related to osteoclast-type giant cells (19). In the present study, two patients manifested expansile osteolytic lesions and one patient with an incomplete sclerotic margin.

CT showed a mass of variable density with indistinct borders. Focal areas of calcification were described as “ice floes” signs, speculating that the possible reason is tumor interstitial calcification, ossification, or residual bone in the tumor (19). A definite fat plane could not be demonstrated in most instances between the tumor and adjacent structures, which is highly suggestive of an infiltrating neoplasm (19). In the present study, two patients manifested “ice floes” sign, one patient with an expansive infiltrative lesion with moderate compression of the neural root and spinal cord. EHE in the spine mainly showed a low T_1_WI signal, a high T_2_WI signal, and a high signal intensity on fat-pressing images (3, 20). Our study confirmed that a lytic lesion has a slight increase in signal intensity on T2-weighted images and a decrease in signal intensity on T_1_WIs. EHE usually has no periosteum reaction, except for the secondary pathologic fracture. However, in this group, 1 case of secondary vertebral compression fracture had no obvious periosteum reaction. A subsequent MRI of the spine revealed a vertebral body compression fracture with central canal stenosis and cord compression, as well as cortical destruction and soft tissue invasion. Most of the mass is located in the epidural and grows across the segment, which may be accompanied by a dural tail sign. EHE can involve the adjacent joints. In this group, 2 patients had EHEs that grew across the vertebral body through the facet joints, the adjacent intervertebral space was slightly narrowed, and no obvious damage was observed in the intervertebral disc. The vertebral artery was slightly narrowed, but no obvious distortion or filling defect was observed. Aflatoon et al. (21) indicated that CT scans revealed an expansile lytic process. All lesions involved the vertebral body, and one was diffuse with spinous process involvement. After the enhancement, it was moderate to obvious enhancement on the CT image, with uneven enhancement; and strong homogeneous enhancement or peripheral enhancement can be observed on enhanced T_1_WIs, with multiple low-signal lesions inside the tumor. The possible reason for the uneven enhancement is tumor interstitial calcification, ossification, interstitial mucoid, or hyaline.

Differential diagnosis of EHE depended on the clinical scenario and the radiological findings; metastasis neoplasm, giant cell tumor of bone, aneurysmal bone cyst, and osteoblastoma being the most common. (i) Giant cell tumor of bone is a biologically benign and locally aggressive tumor that most often affects the epiphyseal and metaphyseal sites of long bones in the young adult population (22). (ii) Bone cyst is a solitary fluid-filled tumor that most often affects the proximal humerus and proximal femur, with no enhancement (10). (iii) Aneurysmal bone cysts are rare skeletal tumors that most commonly occur in the first two decades of life. Radiographic features include a dilated radiolucent lesion typically located within the metaphyseal portion of the bone with fluid-fluid levels visible on MRI (23). (iv) Osteoblastomas are rare benign osteoid-forming tumors that most commonly occur in the second and third decades of life. Radiographic features include mixed lytic and sclerotic lesions with expanded thinned cortices, which predominantly affect the vertebral column and long tubular bones (24).

As with other retrospective studies, the limitations of this study include a relatively small sample size. Furthermore, correlation with age and sex should be assessed in the future.

In conclusion, primary SEHE is a rare low-grade vascular tumor that usually presents as local pain. It should be considered when a mass has an expansile osteolytic lesion with or without a complete or incomplete hardened edge and a distinctive soap bubble matrix with a sclerotic margin like that found in benign vascular tumors with a residual intact/incomplete bone crest. Most of the mass is located in the epidural and grows across the segment, which may be accompanied by a dural tail sign.

## Data Availability Statement

The original contributions presented in the study are included in the article/supplementary material. Further inquiries can be directed to the corresponding author.

## Ethics Statement

The institutional review board approved this retrospective study, and the requirement for written informed consent was waived. This prospective study strictly adhered to HIPAA Privacy rule and was approved by the ethics committees of the institutional review board of the First Affiliated Hospital of Zhengzhou University and Beijing Jishuitan Hospital. The China Biobank project is a multicenter cohort study and has been registered with the US clinical trial database (https://clinicaltrials.gov/ct2/show/NCT03699228; trial identifier: NCT03699228).

## Author Contributions

PL is the primary author. J-BG and PL critically reviewed the paper and revised it. PL, B-BZ, and X-CR performed the database search and literary review. All authors listed have read and approved the article for publication.

## Conflict of Interest

The authors declare that the research was conducted in the absence of any commercial or financial relationships that could be construed as a potential conflict of interest.

## Publisher’s Note

All claims expressed in this article are solely those of the authors and do not necessarily represent those of their affiliated organizations, or those of the publisher, the editors and the reviewers. Any product that may be evaluated in this article, or claim that may be made by its manufacturer, is not guaranteed or endorsed by the publisher.
